# LncRNA LINC00944 Promotes Tumorigenesis but Suppresses Akt Phosphorylation in Renal Cell Carcinoma

**DOI:** 10.3389/fmolb.2021.697962

**Published:** 2021-07-05

**Authors:** Chiheng Chen, Hanxiong Zheng

**Affiliations:** Department of Urology, The Eighth Affiliated Hospital, Sun Yat-sen University, Shenzhen, China

**Keywords:** LINC00944, tumorigenesis, Akt, phosphorylation, RCC

## Abstract

Long non-coding RNA (lncRNA) is a kind of RNA that possesses longer than 200 nucleotides and lacks protein coding function. It was recognized as a junk sequence for a long time. Recent studies have found that lncRNAs are actively functioning in almost every aspect of cell biology and involved in a variety of biological functions. LncRNAs are closely related to a variety of human diseases, especially tumors. Recently, lncRNAs are being increasingly reported in renal cancer. In our study, we identified the expression of lncRNA LINC00944 is significantly elevated in renal cell carcinoma (RCC) tissues and cell lines and high LINC00944 expression is significantly correlated with the tumor stage and prognosis of RCC. The knockdown of LINC00944 by CRISPR/dCas9-KRAB in higher expressing 786-O and 769-P RCC cells could significantly decrease proliferation and migration and also promote phosphorylation of Akt compared with the control group. Our study is the first to report the function of lncRNA LINC00944 in RCC. And we provide clinicopathological and experimental evidence that lncRNA LINC00944 acts as an oncogene in RCC, suggesting that targeting lncRNA LINC00944 expression might be a promising therapeutic strategy for the treatment of RCC.

## Introduction

Renal cell carcinoma (RCC), referred to as renal cancer for short, accounts for about 85% of primary renal malignancies, ranking the third among urinary system tumors, and accounts for about 2–3% of all adult malignancies (Lopez-Beltran et al., 2006; Siegel et al., 2019). In recent years, the global incidence of RCC has been gradually increasing, with about 209,000 new cases and 102,000 deaths per year (GUPTA et al., 2008). Although diagnostic techniques continue to improve, renal cancer treatment has not progressed. The main reason for this is that, in addition to early resection, renal cancer is not sensitive to other types of treatments, such as radiotherapy, chemotherapy, and endocrine therapy, so the treatment effect is not obvious. Postoperative treatment cannot reduce the metastasis rate of renal cancer, and immunotherapy is only effective for 15–20% of patients (Bukowski, 2000; Negrier et al., 2000). However, about 50% of patients with renal cancer were already in the advanced stage when they first visited the doctor, almost 40% of patients showed recurrence or metastasis after surgery, and the three-year survival rate of patients without any treatment was less than 5% (Mulders et al., 1997). At present, researchers are concerned about finding out some new therapeutic methods to improve the therapeutic effect of renal cancer, reduce postoperative recurrence and metastasis, and improve the quality of life of patients while conducting surgical treatment as early as possible.

In the past decade, many new types of non-coding RNAs have been discovered, and the important roles of some non-coding RNAs in gene regulatory networks have been revealed (Qi and Du, 2013). The results of a large number of clinical observations and experimental evidence show that there is a close relationship between the occurrence of renal clear cell carcinoma development and the long chain of non-coding RNAs (lncRNAs). LncRNA is considered important in epigenetics research, especially in life science research (Tang et al., 2013), and is also one of the most popular areas of current research. LncRNA is non-coding RNA with a length of more than 200 nucleotides, including non-coding small RNA, interfering small RNA, PIWI-interacting RNA, nucleolar small RNA, and nuclear small RNA. One of the clear characteristics of lncRNA is the acquisition of secondary and tertiary three-dimensional structures, which are mainly dependent on Watson–Crick base pairing (Geisler and Coller, 2013; Mercer and Mattick, 2013; Novikova et al., 2013). This structure enables it to perform both RNA-related functions based on nucleic acid complementarity and protein-like functions based on spatial conformations (Ørom et al., 2010; Long et al., 2017).

Approximately 2,000 lncRNAs are abnormally expressed in renal cell carcinoma (Malouf et al., 2015). These lncRNAs are characteristic in RCC and are considered to play an important role in the activation of the HIF pathway and are involved in a variety of carcinogenic mechanisms. Zhang et al. (2015) verified this in renal cancer tissues and cell lines and found that compared with paratumoral tissues and normal renal tubular epithelial cell lines, MALAT-1 was highly expressed in renal cancer tissues and renal cancer cell lines and the high expression was correlated with prognosis. Subsequently, Hirata et al. (2015) also reported that MALAT-1 was highly expressed in renal cancer tissues and the high expression of MALAT-1 could promote the expression of EZH2, thus promoting tumor epithelial-to-mesenchymal transition (EMT) and further promoting the progression of renal cancer. Pei et al. (2014) found that HOTAIR was closely related to the metastatic ability of renal cancer cell lines and curcumin may inhibit HOTAIR, thereby inhibiting renal cancer metastasis. Chiyomaru et al. (2014) found that, in renal cancer tissues and cell lines, the expression of miR-141 was negatively correlated with the expression of HOTAIR. Further studies showed that miR-141 could regulate the expression of HOTAIR by interacting with the immune complex A902.

In this study, through the screening and analysis of multiple databases, we screened the lncRNA LINC00944 with obvious differential expression in renal cancer tissues and paracancerous tissues and verified the high expression of LINC00944 in renal cancer tissues. Then, we knocked down LINC00944 by CRISPR/dCas9-KRAB in 786-O and 769-P RCC cell lines. Cell function experiments have shown that downregulation of LINC00944 can reduce the proliferation and metastasis of renal cancer cells. Moreover, we found there was a relationship between the expression pattern of LINC00944 and TYMP in RCC tissues; this indicated that LINC00944 might regulate TYMP expression in RCC. Finally, we detected the activity of the PI3K/Akt pathway by western blot and found LINC00944 knockdown promoted phosphorylation of Akt in RCC cell lines. Therefore, LINC00944 may be closely related to the proliferation and metastasis of renal cancer and play a role in promoting tumor progression. LINC00944 may become one of the effective therapeutic targets for renal cancer.

## Results

### Long Non-Coding RNA LINC00944 Is Highly Expressed in Renal Cell Carcinoma Tissues and Is Associated With Patient Prognosis

**FIGURE 1:**
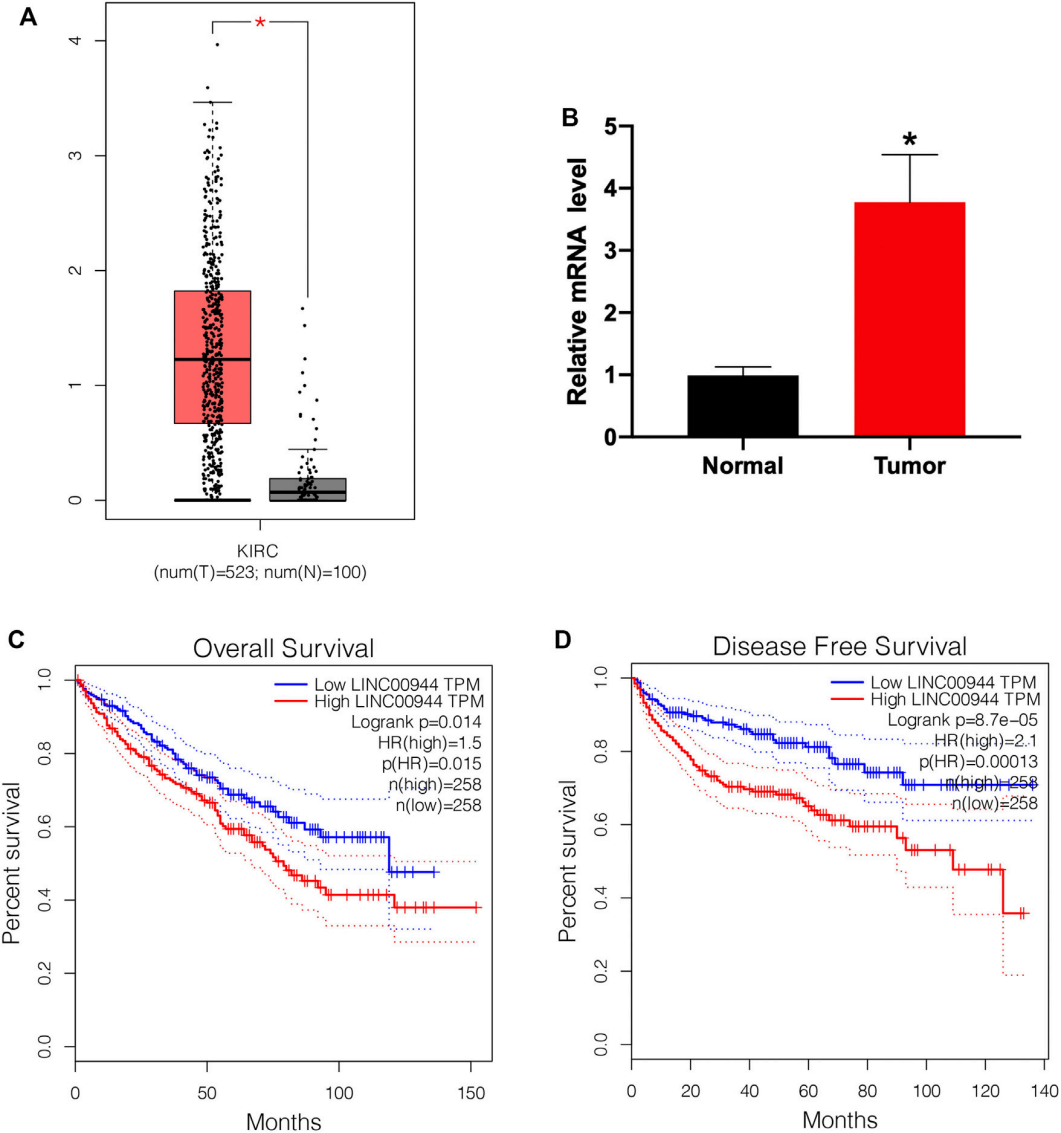
The expression level of lncRNA LINC00944 was significantly increased in RCC patients. **(A)** LINC00944 expression levels in tumor and adjacent tissues from the TCGA database. **(B)** Expression levels of LINC00944 in ten RCC patients detected by qRT-PCR. **(C)** Kaplan–Meier survival curves of LINC00944 in RCC tissues from the TCGA database. **(D)** Disease-free survival curves of LINC00944 in RCC tissues from the TCGA database. *p < 0.05.

To explore the role of lncRNA LINC00944 in the development of RCC, we firstly checked its expression level in RCC tissues and normal tissues from the TCGA database. We found the expression of LINC00944 was significantly increased in RCC tissues compared to normal tissues (Figure 1A). Then, we examined its expression levels in ten RCC patients by qRT-PCR and found it was increased in tumors (Figure 1B). Moreover, Kaplan–Meier survival analysis from the TCGA database showed that the low-expression group of LINC00944 had a longer postoperative survival than the high-expression group (Figure 1C). Furthermore, the disease-free survival analysis showed the same results with the overall survival (Figure 1D).

### Long Non-Coding RNA LINC00944 Is Highly Expressed in Renal Cell Carcinoma Cells and Can Be Knocked Down by dCas9-KRAB

**FIGURE 2:**
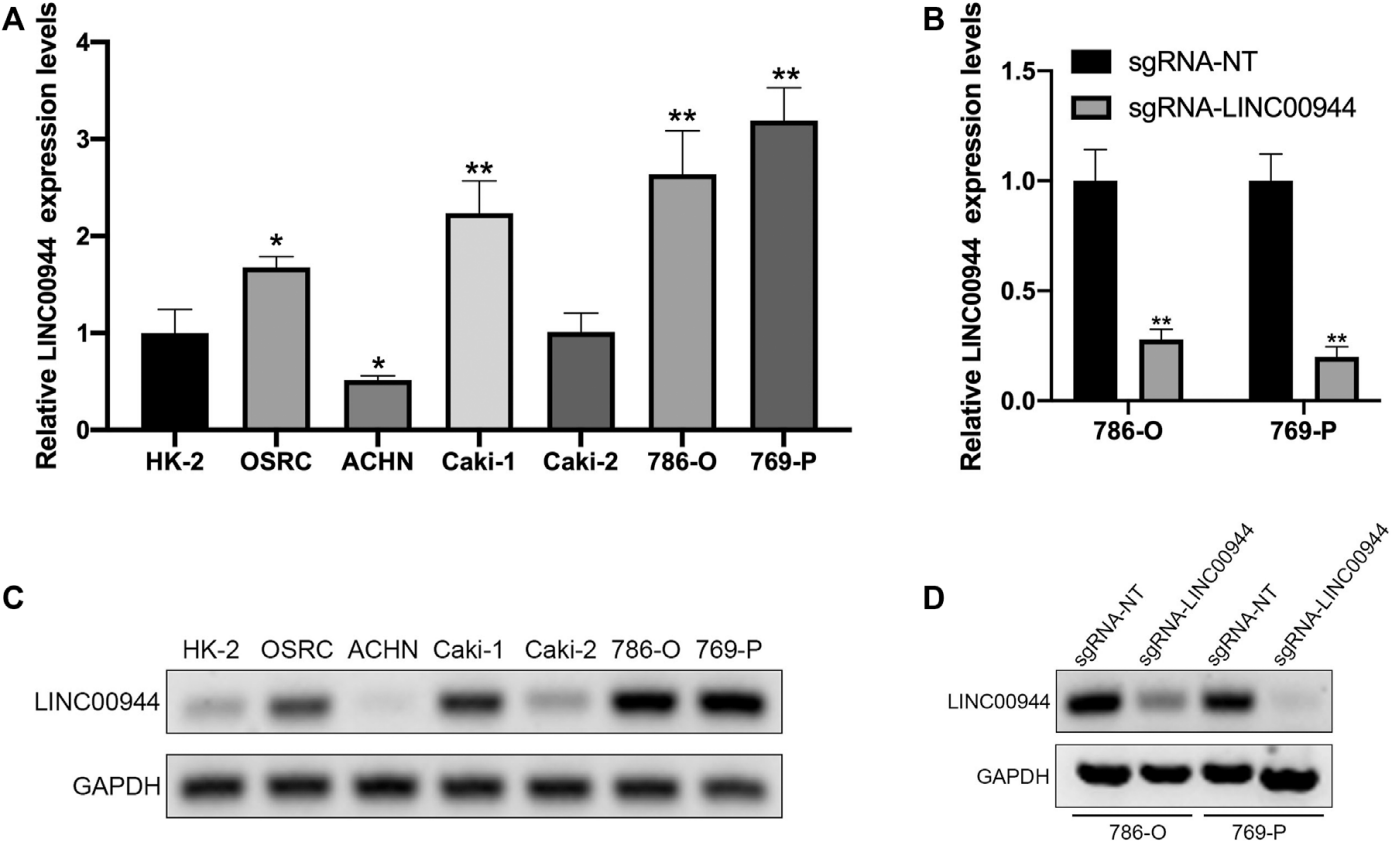
CRISPR/dCas9-KRAB decreased the expression level of LINC00944 in RCC cells. **(A,C)** Expression levels of LINC00944 in RCC cell lines (OSRC, ACHN, Caki-1, Caki-2, 786-O, and 769-P) and normal renal tubular epithelial cell line (HK-2) by qRT-PCR and semi-quantitative RT-PCR. **(B,D)** Expression levels of LINC00944 in 786-O and 769-P RCC cells transfected with dCas9-KRAB and sgRNA-NT or sgRNA-LINC00944 for 24 h by qRT-PCR and semi-quantitative RT-PCR. *p < 0.05; **p < 0.01.

We have confirmed that LINC00944 was highly expressed in RCC tissues, so we used qRT-PCR to detect the expression of LINC00944 in six human renal carcinoma cell lines with different malignant potentials (ACHN, Caki-2, Caki-1, 769-P, 786-O, and OSRC) and one human normal renal tubular epithelial cell line HK-2. The results showed that LINC00944 was highly expressed in OSRC, Caki-1, 786-O, and 769-P RCC cells (Figures 2A,B). To investigate the function of LINC00944 in RCC, we knocked down LINC00944 in 786-O and 769-P RCC cells by dCas9-KRAB, and the expression levels of LINC00944 were decreased (Figures 2C,D).

### Long Non-Coding RNA LINC00944 Promotes the Proliferation in 786-O and 769-P Renal Cell Carcinoma Cells

**FIGURE 3:**
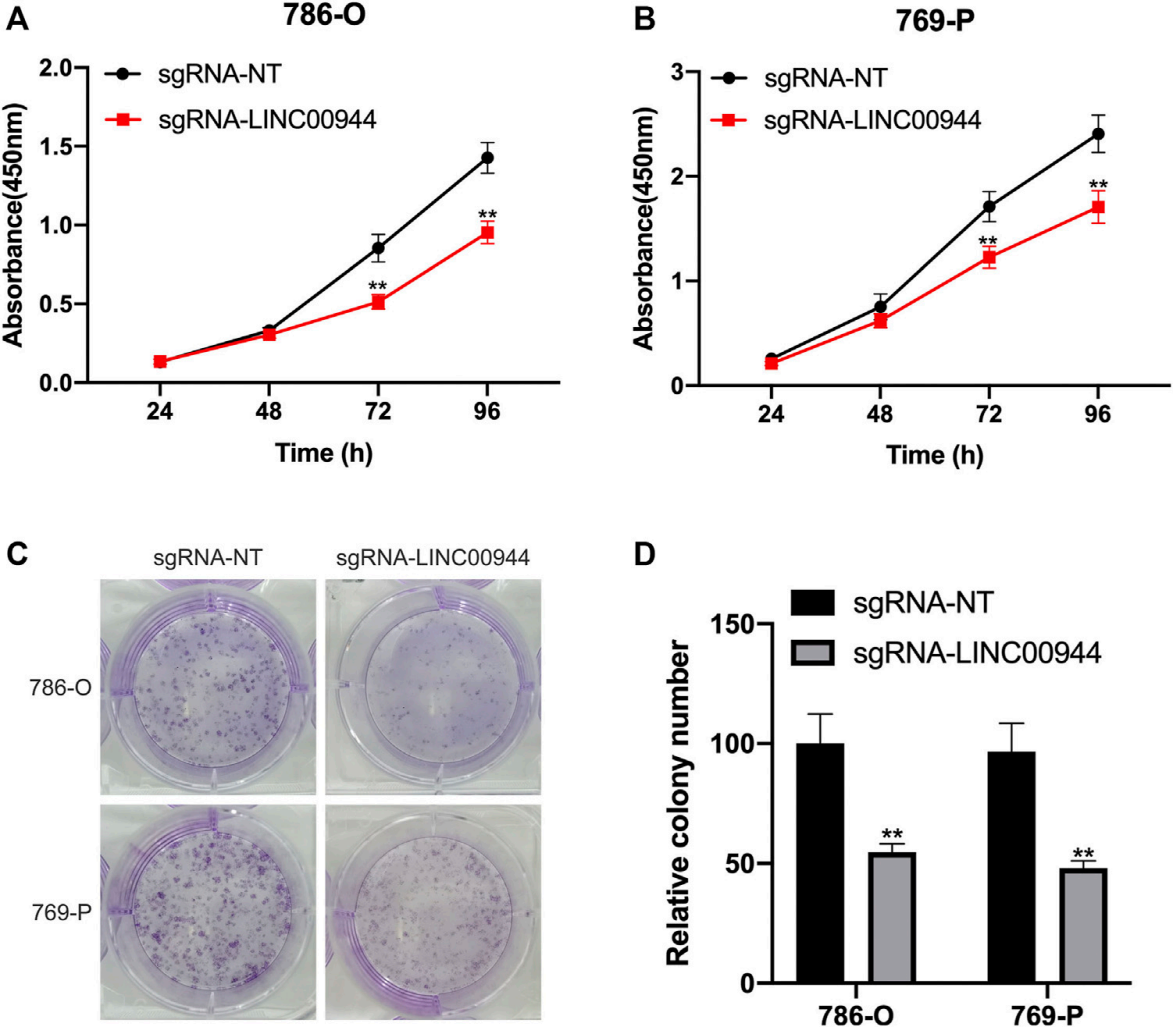
Downregulation of LINC00944 inhibited the proliferation of RCC cells. **(A,B)** CCK-8 assays of 786-O and 769-P RCC cells transfected with dCas9-KRAB and sgRNAs. **(C,D)** Colony formation assays of 786-O and 769-P RCC cells transfected with dCas9-KRAB and sgRNAs. **p < 0.01.

The CCK-8 assay was performed on 786-O and 769-P cells transfected with sgRNAs, respectively. The cell proliferation of 786-O and 769-P groups transfected with sgRNA-LINC00944 was significantly reduced, and the difference gradually increased after the second day compared with the 786-O and 769-P control groups transfected with sgRNA-NT (Figures 3A,B). In order to further verify the effect of LINC00944 on the proliferation ability of RCC cells, the colony formation assay was performed on the cells treated with the above method. The results showed that the number of cell colonies in the 786-O and 769-P groups transfected with sgRNA-LINC00944 was significantly less than that in the 786-O and 769-P control groups transfected with sgRNA-NT (Figures 3C,D).

### Long Non-Coding RNA LINC00944 Promotes the Migration of 786-O and 769-P Renal Cell Carcinoma Cells

**FIGURE 4:**
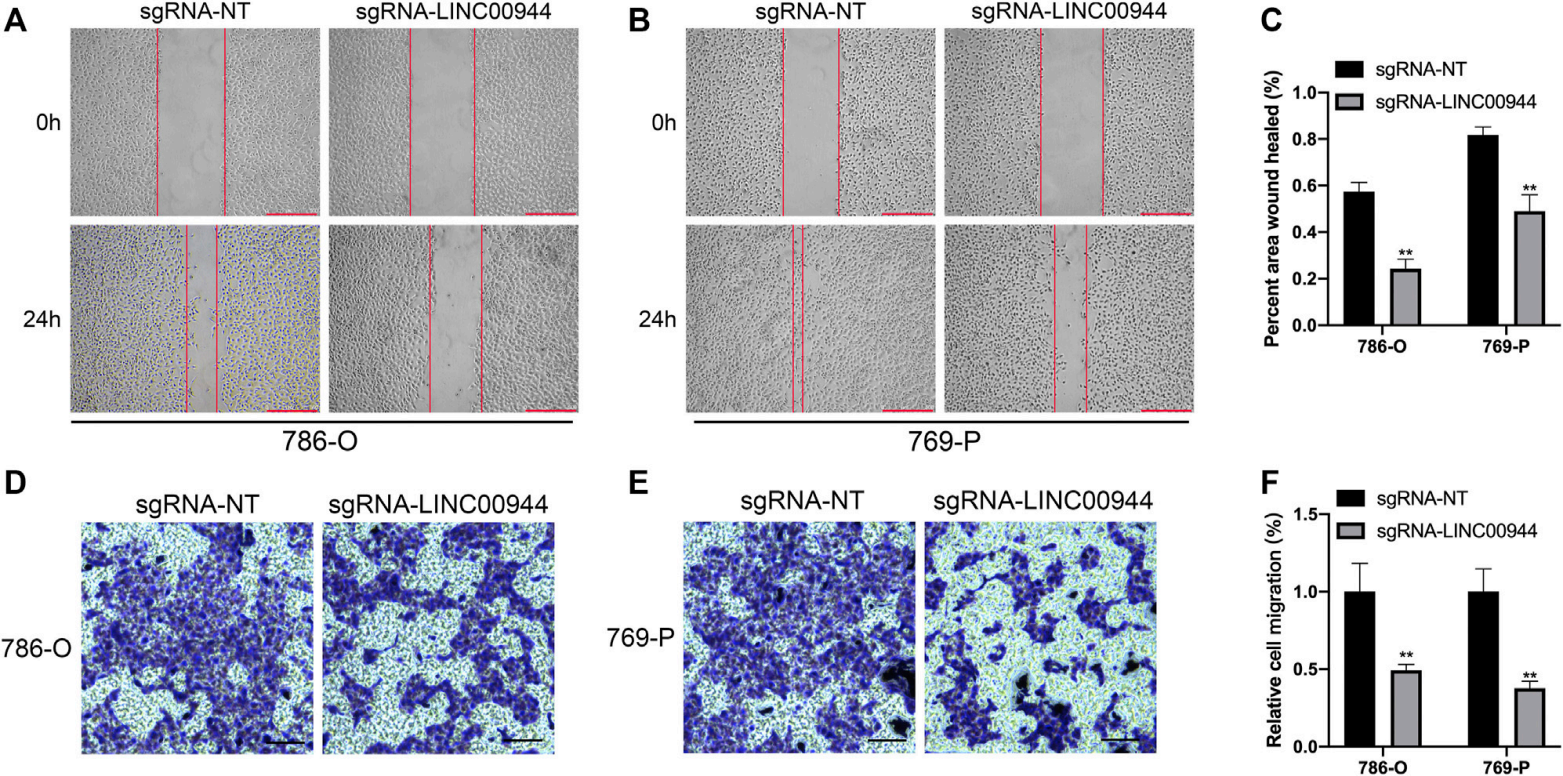
Downregulation of LINC00944 inhibited the migration of RCC cells. **(A–C)** Wound-healing assays for migration ability of 786-O and 769-P RCC cells transfected with dCas9-KRAB and sgRNAs. **(D–F)** Transwell assays for migration ability of 786-O and 769-P RCC cells transfected with dCas9-KRAB and sgRNAs. **p < 0.01.

The migration ability of tumor cells is an important aspect of tumor progression. Therefore, in order to further explore the effect of LINC00944 on the migration ability of 786-O and 769-P RCC cells, we conducted the scratch assay and transwell assay. The ability of scratch healing in the scratch experiment reflects the ability of cell migration. The scratch test results showed that the scratch healing rate of 786-O and 769-P groups transfected with sgRNA-LINC00944 was less than that of 786-O and 769-P control groups transfected with sgRNA-NT after transfection for 24 h (Figures 4A–C). Transwell results showed that compared with the 786-O and 769-P control groups transfected with sgRNA-NT, the 786-O and 769-P groups transfected with sgRNA-LINC00944 inhibited the migration of 786-O and 769-P cells (Figures 4D–F). These results suggest that LINC00944 can promote the migration ability of RCC cells.

### Long Non-Coding RNA LINC00944 Regulates TYMP Expression and Suppresses Akt Phosphorylation

**FIGURE 5:**
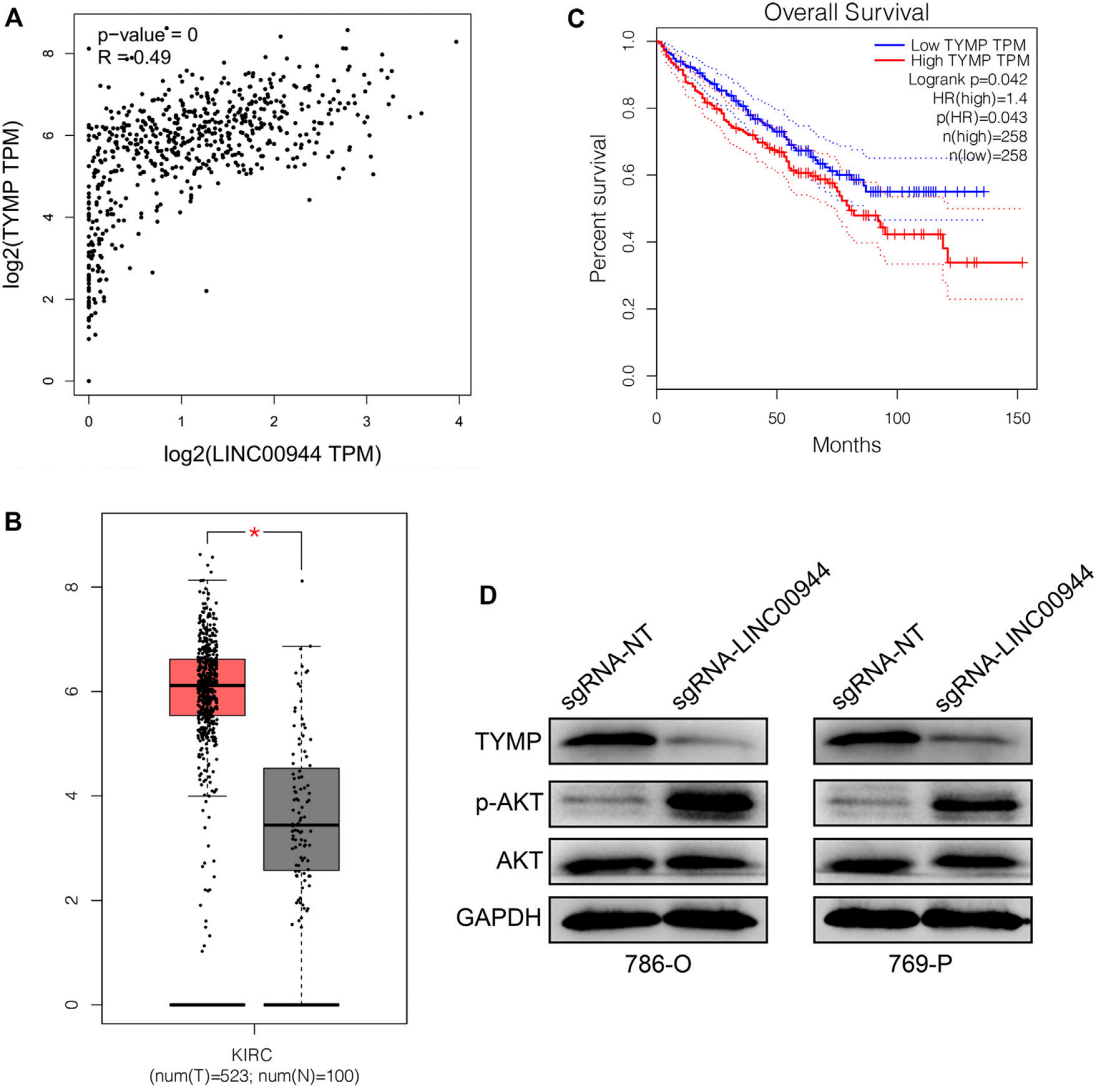
LncRNA LINC00944 regulates TYMP expression and suppresses Akt phosphorylation. **(A)** LINC00944 expression was significantly positively correlated with the expression of TYMP in RCC tissues (data from the TCGA database). **(B)** TYMP expression levels in tumor and adjacent tissues from the TCGA database. **(C)** Kaplan–Meier survival curves of TYMP in RCC tissues from the TCGA database. **(D)** Protein levels of TYMP and Akt phosphorylation in 786-O and 769-P RCC cells transfected with dCas9-KRAB and sgRNAs. *p < 0.05.

From the TCGA database, we investigated the association of LINC00944 expression with the expression of TYMP in a large number of RCC tissues. Expectedly, there was a significantly positive relationship between LINC00944 expression and the expression of TYMP in RCC tissues (Figure 5A). Then, we found the expression level of TYMP was also upregulated in RCC tissues compared to normal tissues (Figure 5B). And Kaplan–Meier survival analysis from the TCGA database showed that the low-expression group of TYMP had a longer postoperative survival than the high-expression group (Figure 5C), which was the same with the expression pattern of LINC00944. Finally, we detected the protein levels of TYMP in 786-O and 769-P RCC cells after knocking down LINC00944. The results showed that the expression levels of TYMP were also decreased after downregulating LINC00944 in RCC cells. Furthermore, the knockdown of LINC00944 significantly promoted Akt phosphorylation in RCC cells (Figure 5D).

## Discussion

Renal cancer is one of the most common malignancies of the urinary system. For early or locally advanced renal cancer, surgical resection is still the most effective treatment, but there are still 20–30% patients with tumor recurrence or metastasis after surgery. Advanced renal cancer is not sensitive to radiation and chemotherapy, and the efficacy of immunotherapy is very limited (Murai and Oya, 2004; Chen et al., 2015). Therefore, it is particularly important and urgent to explore the mechanism of renal cancer development and metastasis and to find a reliable therapeutic target. It is of great significance to clarify the mechanism of renal cancer development and metastasis and to find suitable therapeutic targets for the treatment of the disease. Designing drugs for key targets can reduce drug development effort, and related drugs tend to be more effective and have lower adverse reaction rates. A good example is targeted therapy for renal cancer, where researchers have developed drugs that target the mechanisms involved in angiogenesis in renal cancer such as vascular endothelial growth factor tyrosine kinase inhibitors (TKIs) sunitinib, axitinib, pazopanib, and so on.

Recent studies have found that long non-coding RNAs play important roles in a variety of biological processes, such as cell growth, apoptosis, differentiation, and metastasis. Similarly, long non-coding RNAs are thought to be involved in tumor progression and metastasis. In fact, long non-coding RNAs can affect the transcription and translation of coding genes through a variety of pathways, such as chromosome remodeling, transcriptional activation or inhibition, protein inhibition, and post-transcriptional modification. Fachel et al. (2013) found that a series of intergene lncRNAs can affect gene expression through cis and trans effects, thereby regulating the occurrence and development of tumors. For example, HOTAIR can recruit PRC2 and LSDL complexes to specific sites through trans-regulation, resulting in methylation or demethylation of specific genes, thereby mediating tumor genesis and metastasis (Wu et al., 2014). LncRNA can also play a role by acting as a competitive binding miRNA between ceRNA and mRNA (Zhou et al., 2014). For example, Yuan et al. (2014) found that, in the occurrence of liver cancer, lncRNA-ATB could competitively bind to miRNA-200 with ZEBL and ZEB2, thereby upregulating the expression of ZEBL and ZEB2, promoting the process of tumor EMT, and thus promoting the metastasis of liver cancer. However, the mechanism of action of long non-coding RNA in renal cancer is still unclear, which needs further exploration and research.

In this study, we searched renal cancer–related datasets through the TCGA database and screened lncRNAs, and we found that lncRNA LINC00944 was significantly upregulated in renal cancer tissues. The expression level and clinical characteristics of lncRNA LINC00944 were analyzed, and it was found that the high expression of lncRNA LINC00944 was related to the tumor stage. The results of survival analysis showed that compared with the low-expression group, the high-expression group had shorter postoperative survival time. In order to further investigate the biological function of lncRNA LINC00944 in RCC, we constructed 786-O and 769-P cell lines capable of knocking down lncRNA LINC00944 by using CRISPR/dCas9-KRAB plasmid transfection. The results showed that compared with the control group transfected with sgRNA-NT, the proliferation and migration abilities of 786-O and 769-P cells transfected with sgRNA-LINC00944 were significantly decreased, suggesting that knockdown of lncRNA LINC00944 expression could inhibit the proliferation and metastasis of RCC. Furthermore, we found lncRNA LINC00944 could regulate TYMP expression and suppress Akt phosphorylation in RCC cells.

In summary, this study is the first to report the function of lncRNA LINC00944 in RCC. Through clinicopathological data and *in vitro* cell experiments, it is confirmed that lncRNA LINC00944 plays an oncogenic role in RCC. Knocking down the expression of lncRNA LINC00944 could inhibit cell proliferation and migration. These experiments suggested that lncRNA LINC00944 could serve as a new therapeutic target for RCC therapy.

## Methods

### Cell Culture

The human RCC cell lines 786-O, 769-P, ACHN, OSRC, Caki-1, and Caki-2 and human renal tubular epithelial cell line HK-2 used in this study were all preserved by the Eighth Affiliated Hospital of Sun Yat-sen University. In *in vitro* cell culture and experiment, 786-O, ACHN, and OSRC cell lines were cultured in RPMI-1640 medium (Hyclone), Caki-1 cells were cultured in high-glucose medium (Hyclone), and Caki-2 cells were cultured in McCoy 5A medium (Hyclone). All the above media were supplemented with 10% fetal bovine serum (FBS; Gibco); all cells were incubated in a 37°C chamber containing 95% air and 5% carbon dioxide. In addition, trypsin used in cell culture came from Beijing Solarbio Biological Technology Co., Ltd., and pipette heads, centrifuge tubes, petri dishes, and cryopreserved tubes were all from Coming Company. The other main instruments are used continuously in the laboratory all year round with good performance.

### Plasmid Transfection

The plasmids used in this study were synthesized and provided by Beijing Yingmaoxiang Technology Co., Ltd. According to the instructions, dCas9-KRAB and sgRNA-NT or sgRNA-LINC00944 were transfected into 786-O and 769-P cell lines using Lipofectamine 2000 (Invitrogen, United States), and the cells after transfection for 24–48 h were used for the next experiment. After treatment, 786-O and 769-P cell lines were divided into two groups: the experimental group (transfected with sgRNA-LINC00944) and the control group (transfected with sgRNA-NT).

### Cell Proliferation Assay

The CCK-8 assay is used to reflect cell proliferation. The untreated cells (about 1 × 10^5^ 786-O cells and 2 × 10^5^ 769-P cells) were spread into a 60 mm plate and cultured for 24 h; then, the plasmid was transfected into the cells according to the above method. 24 h after transfection, 1,000 cells were spread into 96-well plates, and 100 μL of 10% serum was added to each well, followed by incubation in a 37°C incubator. The 96-well plates to be measured were removed at 0, 24, 48, 72, and 96 h after plate placement, and 20 μL buffer was added to each well (Promega, United States). After further incubation for 2 h, the absorbance values of each well at 490 nm were read using a 96-well plate enzyme-linked immunoreader (BioTek Instruments, United States). The abscissa of absorbance value of cells treated in each period was time, and the ordinate was the absorbance value to draw the cell proliferation curve. The experiment was repeated three times with three replicates each time.

### Colony Formation Assay

786-O or 769-P cells were inoculated into six-well plates and transfected with plasmids, respectively. After 36 h of transfection, cells in each group were collected and counted by suspension. Cells in each group were inoculated into six-well plates containing 5 ml medium with 10% fetal bovine serum, with 500 cells in each plate. After 14 days, they were taken out (the solution could be changed 1–2 times), fixed with formaldehyde for half an hour, and stained with crystal violet. The number of clones was counted under high magnification (the number of cells per clone should be greater than 50). Each experiment was repeated three times, and three replicates were set for each sample.

### Cell Scratch Assay

The scratch test was used to detect cell migration. 2 × 10^5^ 786-O or 769-P cells were inoculated into a six-well plate, and 3 ml medium containing 10% serum was added. After the cells were fused into a layer of monolayer cells and covered the bottom of the dish, a 200 μL sterile yellow spear head was used to draw a straight line at the diameter of the dish at a constant speed. The fresh serum-free medium was changed and incubated in a warm box. Photographs were taken at the same location in the same dish 0 and 24 h after scratches. The scratch experiment was repeated three times, and three points were selected from the same dish for measurement each time.

### Transwell Assay

After 48 h of cell transfection, the supernatant was discarded and suspended to 10,000 cells/mL, and 200 μL of suspension was added into the upper chamber of the 24-well plate. 500 μL culture medium containing 5% fetal bovine serum was added in the lower chamber, followed by incubation in a warm box for 8–12 h. The upper chamber and the medium were removed, the upper cells and matrix were wiped out with a clean cotton swab, and the migratory cells were retained on the lower surface of the upper chamber. The cells were fixed with formaldehyde for half an hour, stained with crystal violet for 1 h, rinsed with water for a few seconds, and placed in an oven at 80°C for 30 min, and the number of cells that migrated to or invaded the lower surface was counted under a microscope. Each experiment was repeated three times.

### RNA Isolation and Quantitative Real-Time PCR

Total RNA of the cell lines was extracted using Trizol reagent (Invitrogen, CA) and reverse transcribed into cDNA using the One-Step RT-PCR Kit (Beijing Transgen Biotechnology Co., Ltd.) according to the instructions. The expression level of lncRNA was quantitatively detected using a 7500 detection system (Applied Biosystems, Foster City, CA), and the SYBR reagent was purchased from Beijing Transgen Biotechnology Co., Ltd. The reaction system of qRT-PCR was as follows: a 20 μL system consisting of 0.5 μL cDNA, 0.8 μL upstream and downstream mixed primers, 10 μL mix containing SYBR Green, 0.4 μL ROX mixture, and 8.3 μL deionized water. The cycle parameters were as follows: one cycle at 95°C for 60 s and then 40 cycles at 95°C for 5 s and 60°C for 34 s.

### Western Blotting

Proteins in cells were extracted according to the instructions of the cell protein extraction kit. According to the instructions of the Western Blot Kit, 80 µg protein was added to each well for SDS-PAGE electrophoresis. After cell electrophoresis, the protein was transferred to the PVDF membrane. The PVDF membrane was sealed with 5% skimmed milk powder, and the primary antibody was incubated in a shaker at room temperature for 2 h. The films were washed with TBST for 3 × 5 min, incubated with a corresponding dilution ratio of secondary antibody for 60 min, and washed with TBST for 3 × 10 min. Enhanced chemiluminescence was performed with GAPDH as the internal reference, and the gray optical density of the relative bands was analyzed with ImageJ software.

### Statistical Analysis

Each experiment was repeated three times, and the data were expressed in terms of mean ± standard deviation. All data were analyzed using SPSS 16.0 (SPSS, Chicago, United States), and graphs were drawn using GraphPad Prism 6 (GraphPad Software, San Diego, CA, United States) and Adobe Illustrator (Adobe, United States). *t*-test, ANOVA, Fisher’s exact test, chi-square test, and Wilcoxon test were used to determine whether the differences between groups were statistically significant. *p* < 0.05 was considered statistically significant.

## Data Availability

The original contributions presented in the study are included in the article/Supplementary Material, and further inquiries can be directed to the corresponding author.
