# Supplementary figures and images for: LncRNA LINC00944 Promotes Tumorigenesis but Suppresses Akt Phosphorylation in Renal Cell Carcinoma
Source: Front Mol Biosci. 2021 Jul 5;8:697962. doi: 10.3389/fmolb.2021.697962 (PMC8287069; doi:10.3389/fmolb.2021.697962)

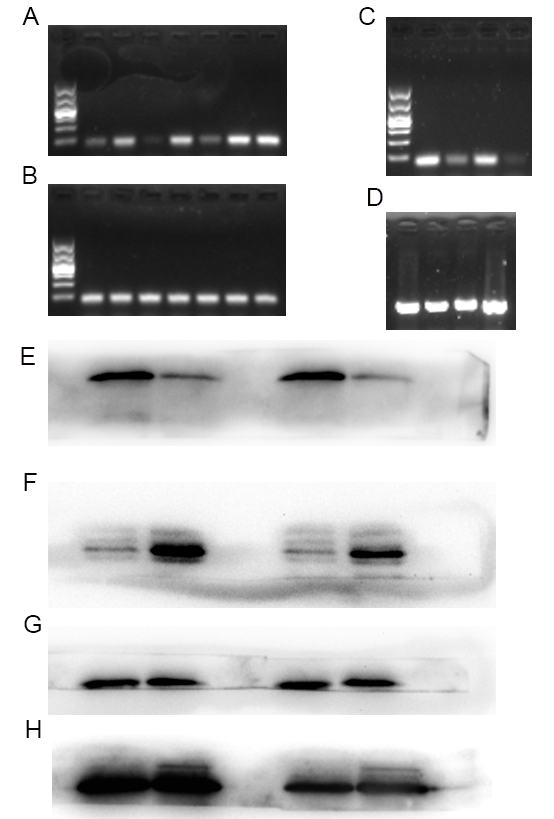

Supplement: Supplementary file 1 [file Image1.TIF]
